# Do existing mechanisms contribute to improvements in care coordination across levels of care in health services networks? Opinions of the health personnel in Colombia and Brazil

**DOI:** 10.1186/s12913-015-0882-4

**Published:** 2015-05-29

**Authors:** Ingrid Vargas, Amparo Susana Mogollón-Pérez, Pierre De Paepe, Maria Rejane Ferreira da Silva, Jean Pierre Unger, María Luisa Vázquez

**Affiliations:** Health Policy and Health Services Research Group, Health Policy Research Unit, Consortium for Health Care and Social Services of Catalonia, Avenida Tibidabo, 21, 08022 Barcelona, Spain; Faculty of Medicine and Health Sciences, Universidad del Rosario, Carrera 24, No. 63C -69, 11001 Bogotá, Colombia; Prince Leopold Institute of Tropical Medicine, Nationalestraat, 5, 2000 Antwerpen, Belgium; Universidade de Pernambuco, Avenida Agamenon Magalhães, s/n, 50100-010 Recife, Brazil

**Keywords:** Care coordination mechanisms, Care integration, Referral letters, Patient referral systems, Integrated delivery systems, Integrated healthcare networks, Brazil, Colombia, Qualitative research, Health personnel’s views

## Abstract

**Background:**

The fragmentation of healthcare provision has given rise to a wide range of interventions within organizations to improve coordination across levels of care, primarily in high income countries but also in some middle and low-income countries. The aim is to analyze the use of coordination mechanisms in healthcare networks and its implications for the delivery of health care. This is studied from the perspective of health personnel in two countries with different health systems, Colombia and Brazil.

**Methods:**

A qualitative, exploratory and descriptive-interpretative study was conducted, based on a case study of healthcare networks in two municipalities in each country. Individual semi-structured interviews were conducted with a three stage theoretical sample of a) health (112) and administrative (66) professionals of different care levels, and b) managers of providers (42) and insurers (14). A thematic content analysis was conducted, segmented by cases, informant groups and themes.

**Results:**

The results show that care coordination mechanisms are poorly implemented in general. However, the results are marginally better in certain segments of the Colombian networks analyzed (ambulatory centres with primary and secondary care co-location owned by or tied to the contributory scheme insurers, and public providers of the subsidized scheme); and in the network of the state capital in Brazil. Professionals point to numerous problems in the use of existing mechanisms, such as the insufficient recording of information in referral forms, low frequency and level of participation in shared clinical sessions, low adherence to the few available clinical guidelines and the lack of or inadequate referral of patients by the patient referral centres, particularly in the Brazilian networks. The absence or limited use of care coordination mechanisms leads, according to informants, to the inadequate follow-up of patients, interruptions in care and duplication of tests. Professionals use informal strategies to try to overcome these limitations.

**Conclusions:**

The results indicate not only the limited implementation of mechanisms for coordination across care levels, but also a limited use of existing mechanisms in the healthcare networks analyzed. This has a negative impact on coordination, efficiency and quality of care. Organizational changes are required in the networks and healthcare systems to address these problems.

## Background

Concerns regarding the fragmentation of healthcare provision, especially in the care of chronic conditions [1], have sparked a wide variety of interventions for the improvement of care coordination, primarily in high-income countries, but also in some middle and low-income countries [2, 3]. Firstly, there have been interventions at the *macro-level*: policies and regulatory mechanisms that foster the coordination of health care [4, 5]. Secondly, at the *meso-level*: the promotion of organizational initiatives to integrate healthcare delivery, such as the *integrated healthcare network* (IHN), which is defined as a network of organizations that provides or arranges to provide a coordinated continuum of health services to a defined population and is willing to be held clinically and fiscally accountable for the outcomes and the health status of the population served [3, 6]. Lastly, there have also been interventions at the *micro-level*: the introduction of a single coordination mechanism or a combination of mechanisms in a comprehensive program to improve clinical management [4].

This article, which forms part of a wider study [7–11], focuses on the use of mechanisms for coordination between care levels in the healthcare networks of Colombia and Brazil.

### A typology of care coordination mechanisms

*Care coordination* is defined here as the harmonious connection of the different services and activities needed to provide care to a patient throughout the care continuum in order to achieve a common objective without conflicts [12]. Three interrelated types can be distinguished: *information coordination* or the transfer and use of the patient clinical information needed to coordinate activities between providers; *clinical management coordination* or the provision of care in a sequential and complementary way [13], and *administrative coordination*, or the coordination of patient access across the continuum of services according to their needs, an aspect less frequently conceptualised by scholars [14].

Health organizations may opt for different types of mechanisms to coordinate care depending on the basic processes on which they are based [12, 15, 16] (Table 1): 1) Based on programming: *standardization* mechanisms, useful for those situations which can be anticipated–and therefore standardized–and do not necessarily require a rapid response. Coordination is achieved by specifying skills, processes (e.g. clinical guidelines) or outcomes in advance. 2) Based on feedback: a) *direct supervision,* generally used to coordinate activities which do not involve high levels of uncertainty; this is achieved by making one person responsible for the work of the rest, giving instructions and controlling the activities of their staff (e.g. program managers); b) *mutual adjustment* mechanisms, useful when the volume of information to be processed is high and the activities are highly specialized and interdependent (e.g. liaison positions, multidisciplinary cross-level teams). Coordination is achieved through direct contact between individuals in order to solve the problem at the same level at which the information was generated [12].

**Table 1:** Types of mechanisms targeted to improve care coordination

Coordination basis	Theoretical coordination mechanisms		Care coordination mechanisms
Programming	Standardization of skills		Expert system: continuing medical training, alternatives to traditional consultations (case review, clinical sessions, etc.)
	Standardization of work processes		Clinical guidelines, healthcare maps, pharmacological guides, discharge planning;
			Patients’ referral protocols and pathways;
			Shared appointment system;
			Action planning system.
	Standardization of outputs		Healthcare maps;
			Performance monitoring systems.
Feedback	Direct supervision		Program or process manager.
	Mutual adjustment	Informal communication	E-mail, post, web, phone, informal meetings.
		Liaison mechanisms	Multidisciplinary, interdisciplinary and transdisciplinary healthcare teams;
			Liaising job posts: case manager and patient referral centres;
			Permanent committees: cross-level management committee;
			Integration manager: care manager, cross-field manager;
			Matrix design.
		Integrated information system	Referral and counter-referral form;
			Clinical information systems.

Source: Adapted from Terraza et al. [12] based on Mintzberg [15] and Galbraith [16]

Most of the available literature on care coordination interventions is based on research conducted in high-income countries, focusing on a specific clinical condition or coordination mechanism, mainly of the clinical management type, and implemented within certain settings, not in the health services network as a whole [2, 17, 18]. Analyses look at the effectiveness of coordination interventions by measuring their impact on the use of services (e.g. hospital readmissions), costs or outcome indicators (e.g. mortality or changes in patients’ health status) [2, 17–21]. Such analyses fail to relate their results to the use of mechanisms or their effects on health care delivery from the perspective of health professionals, who are ultimately the users of these mechanisms [17]. The few qualitative studies available on the use of a specific coordination mechanism-generally the electronic medical record [22–24], clinical guidelines or care pathways for a specific pathology [25–27]-tend to signal a frequent lack of awareness of the mechanism on the part of health professionals [23, 25, 26] and its insufficient or inadequate use [23, 25]. These problems are linked to two types of factors: firstly, individual factors, such as a lack of ability or knowhow to use it [26, 28], resistance to its implementation [24, 27, 28] or prior negative experiences with similar mechanisms [27, 29]; and secondly, organizational factors, such as inadequate resources and working conditions for its use [23, 24, 26–28].

### Policies to foster healthcare coordination in Colombia and Brazil

Poor care coordination is considered to be one of the main obstacles to attaining effective healthcare outcomes in many Latin American countries, leading to difficulties in access to care, poor technical quality, discontinuity of care and inefficiency in the use of scarce resources [3]. Thus many countries in the region, including Colombia and Brazil, have promoted the development of integrated healthcare networks (IHN).

The type of healthcare network promoted by policies in Colombia and Brazil differs according to the organization of the health system. Colombia has its General System of Social Security in Health (SGSSS), a managed competition model made up of two different insurance schemes: contributory for formal sector employees and those able to pay, and subsidized for the low income population [30]. The SGSSS envisages *enrolment-based healthcare networks*, organized mainly by private for-profit insurers (EPS and EPS-S) that receive a capitation payment per enrolee to cover a benefits package. Insurers may provide services directly through mergers or strategic alliances with providers–a strategy restricted to the contributory scheme and limited to a maximum of 30 % of the insurer’s healthcare expenses [31]-or by contracting private and public healthcare providers. Insurers establish different payment mechanisms for the services contracted (e.g. per capita payment, case-based reimbursement, fee-for services, etc.) [30, 32]. The uninsured population, 8.3 % [33], receives care in public healthcare networks, which are delimited geographically and organized by regional and local health authorities.

Brazil created the Unified Health System (SUS), conceived as a national health system with universal coverage, financed by taxes and decentralized into the different levels of government: federal, state and municipal [34]. It envisages the organization of health services into *regional-based networks* at the supra-municipal level (*regiões*), made up of public and contracted private providers (profit or non-profit). The municipalities, in coordination with their states, are responsible for organizing the network of services for their populations, providing primary care and guaranteeing specialist care through direct provision or agreements (“*pactos”*) with other municipalities [35]. The taxes to finance the SUS are levied mostly at the federal level and transferred to specific municipal and state funds depending on the health services they manage: for primary care and drugs the allocated budget is based on capitation, and for secondary care, there is a prospective payment based on activity [35].

In both countries, care is organized by levels of complexity, with primary care as the entry point and the patient’s care coordinator, and the secondary level in a supporting role [36, 37]. Policies drawn up in Brazil in recent years envisage the implementation of a wide variety of coordination mechanisms for clinical management between care levels (shared clinical guidelines and joint case reviews), along with other strategies for shared care (case management and disease management programs) [36]. In Colombia, the implementation of this kind of mechanism is included in the norms for the accreditation and evaluation of providers, but only as individual quality criteria, not for coordination with the healthcare providers of other levels [38–40]. In both countries the use of patient referral centres is proposed for the coordination of access between levels, although in Colombia this is only the case for emergency care [30, 32, 35, 38, 41]. Evaluations of the use of mechanisms for coordination between care levels are scarce in these countries. In fact, in Colombia there are none, and although in Brazil there are some studies, they are usually limited-with a few exceptions [42]-to the analysis of a specific coordination mechanism, such as the implementation process of the ‘expert system’ (*apoio matricial)*-joint training, joint case review, etc.-in the area of mental health [43, 44], or the recording of information in referral letters [45, 46]. Very few delve deeper into the use of mechanisms or their effects on health care delivery from the point of view of health professionals [44, 47].

This article aims to analyze the use of mechanisms for coordination between health care levels and the implications for health care from the perspective of health personnel in the healthcare networks of Colombia and Brazil.

## Methods

### Study design and area

A qualitative, exploratory and descriptive-interpretative study was carried out based on case studies of healthcare networks in Colombia and Brazil. A case study approach was designed to provide extensive information on the subject of study–the use of care coordination mechanisms-in countries with different healthcare systems, based on individual cases [48, 49].

The study was carried out in two areas of Colombia: the locality of Kennedy (Bogotá, D.C.) and the bordering municipality of Soacha; and three areas in Brazil: the state capital of Pernambuco, Recife, an adjacent municipality, Paulista, and a municipality of the state’s interior, Caruaru. The areas were selected for being densely populated urban spaces with a high proportion of the population belonging to the low or medium-low socioeconomic strata.

### Sample of informants

A theoretical sample was selected in three stages.

Stage I: Selection of cases, which were defined as the network of health services responsible for the care of the enrolled/resident population in the study areas. These were selected applying the following criteria: networks which provide a minimum of primary and secondary care for a defined population. In Colombia: i) insurers (EPS/EPS-S) with their own or contracted network of providers, ii) of both schemes: contributory and subsidized; and in Brazil: i) municipalities with full management of the health system or of extended primary care, ii) with different proportions of the population covered by the Family Health Program [Programa de Saúde da Família] (PSF). In Colombia, all the insurers operating in the study areas were contacted and invited to participate by means of a letter addressed to the manager. Most of them refused to participate in the study (22 out of 27). Four networks were finally selected in Colombia (one per insurance scheme in each area) and three in Brazil, corresponding to the public healthcare network in each area.

Stage II: Selection of health providers of different care levels (primary, secondary and tertiary care) providing care for the population of the study areas; in Colombia, with different levels of integration with the insurer (owned or contracted), and in Brazil, with different geographical access to secondary/tertiary care. The contributory networks in Colombia included *ambulatory care centres*, which offer primary and outpatient secondary care in the same location.

Stage III) Selection of informants from different groups to ensure variation in discourse: a) health professionals of the selected providers with at least six months’ experience, and administrative professionals of providers and insurers working in support services related to the coordination of patient access across care levels (reception, user service desk, patient referral centres, etc.) with at least six months’ experience; and b) managers (chief directors, heads of department or middle managers) of providers and insurers. For the selection of informants, an institutional contact provided a list of possible candidates who met the above criteria, and the researchers selected the informants from the list. In one of the contributory scheme networks of Colombia, all the administrative personnel and managers of the insurer contacted refused to participate (Table 2). The final sample size was between 26 and 40 informants per network, depending on when information saturation was reached.

**Table 2:** Final composition of the informant sample

Study networks		Healthcare professionals		Administrative personnel		Managers		Total
		I level ^(a)^	II, III level ^(a)^	Insurers	Providers	Insurers	Providers	
Soacha	Network 1-S	8	7	2	12	5	6	40
	Network 4-C	5	7	0^(b)^	12	0^(b)^	3	27
Bogotá	Network 2-S	7	10	1	8	4	6	36
	Network 3-C	11	8	4	9	5	2	39
Recife	Network 1	10	11	-	6	-	9	36
Paulista	Network 2	8	7	-	7	-	8	30
Caruaru	Network 3	6	7	-	5	-	8	26

^(a)^ I level – Primary care level, II level – Secondary care or medium complexity level, III level – Tertiary care or high complexity level

^(b)^ The invited potential informants refused to participate in the study. S-Subsidized, C-Contributory

### Data collection

Individual semi-structured interviews were conducted using a topic guide with one common section and one specific section for each informant group. The topic guide included awareness of the mechanisms implemented in the network for coordination between care levels, their use and the factors influencing this, and the impact of these mechanisms on coordination and care. Interviews were conducted by the research team in each study country, made up of senior researchers with an in-depth knowledge of qualitative methods, the research topic and the context, as well as junior researchers that were intensively trained to develop the knowledge and skills required and closely supervised by the senior researchers and project coordinators. The interviews, mostly conducted in the workplace, lasted between 1 and 2 hours and were audio-recorded and fully transcribed. The fieldwork took place between October 2009 and February 2011.

### Data analysis and quality of information

A thematic content analysis [50] was conducted using Atlas-ti software. Data were segmented by study case, informant group and themes. The process of category generation was mainly inductive, emerging from the data. Themes were identified, coded, re-coded and classified, identifying common patterns by looking at regularities, convergences and divergences in data, through a process of constant comparisons, going back and forth between data.

In order to ensure quality of data, the information was triangulated. Results of different groups of informants were contrasted with one another and with the literature. Moreover, six analysts worked collaboratively on the analysis, and differences were discussed until an agreement was reached. These analysts had different backgrounds and an in-depth knowledge of qualitative methods, the research topic and the context. Researchers gained awareness of their assumptions and preconceptions through reviewing the literature on the subject, seeking critique from experts in the subject under investigation, and recording and discussing their assumptions throughout the research process.

### Ethical considerations

Conditions of study procedure, risk and benefit evaluation, confidence and privacy, and informed consent were approved by the ethical committees in the participating countries: Clinical Research Ethics Committee, Municipal Institute of Health Care, Spain (Comité Ético de Investigación Clínica, Instituto Municipal de Asistencia Sanitária); Institutional Review Board, Prince Leopold Institute of Tropical Medicine, Belgium; Research Ethics Committee, School of Medicine and Health Sciences, University of Rosario, Colombia (Comité de Ética en Investigación, Escuela Ciencias de la Salud, Universidad del Rosario); Ethics Committee for Research, Integrated Health Centre Amaury de Medeiros, University of Pernambuco, Brazil (Comitê de Ética em Pesquisa, Centro Integrado de Saúde Amaury de Medeiros, Universidade de Pernambuco); National Research Ethics Committee, National Health Council, Ministry of Health, Brazil (Comissão Nacional de Ética em Pesquisa, Conselho Nacional de Saúde, Ministérios de Saúde, Brazil). In addition, confidentiality agreements were signed with all participating institutions. Free and written informed consent was obtained from every interviewee. The recordings and transcripts were coded in such a way that the individual origin could not be identified, and were then stored in a suitable manner.

## Results

### What coordination mechanisms are health personnel aware of in the health service networks?

In both countries, informants mainly highlight the existence of mechanisms for information coordination and for administrative coordination of access across care levels (Table 3), but with important differences between networks: in the subsidized scheme networks of Colombia, the informants from the Bogota network make reference to a greater variety of mechanisms, but only for specific programs (maternal-perinatal and some chronic pathologies) and only in the case of public healthcare providers. The situation is similar in the state capital network in Brazil (Network 1). In the contributory scheme networks of Colombia, in addition to information transfer mechanisms, informants identify a series of coordination mechanisms for clinical management, mainly related to standardization, but only in ambulatory care centres and for certain external specialists contracted by the networks. No shared care strategies were identified that combine different care coordination mechanisms in a comprehensive program. Moreover, the entity responsible for the implementation of mechanisms varied according to the healthcare network: in the Colombian contributory networks they are introduced primarily by the insurers, while in the Colombian subsidized networks and in Brazil, they are mainly introduced by the municipal health department.

**Table 3:** Mechanisms of care coordination identified by the informants in the networks in Colombia and Brazil

Tipe of coordination	Colombia				Brazil		
	Red 1-S	Red 2-S	Red 3-C	Red4-C	Red1	Red2	Red 3
Information	Referral and counter-referral form	Referral and counter-referral form	Referral and counter-referral form^a^	Referral and counter-referral form^a^	Referral and counter-referral form	Non standardized referral and counter-referral form	Non standardized referral and counter-referral form
	Discharge report	Discharge report	Discharge report	Discharge report	Discharge report	Discharge report	Discharge report
			Shared clinical record^b^	Shared clinical record^b^			
Clinical management			Expert system *(“Primary group”)* ^b^	Expert system *(“staff”)* ^b^	Expert system *(“apoio matricial”)*		
	Clinical guidelines (specific processes)	Clinical guidelines (specific processes)	Clinical guidelines^b^	Clinical guidelines^b^			
Administrative	Patient referral centre	Patient referral centre	Patient referral centre (*“24 h telephone line”*)	Patient referral centre (*“Provider line”*)	Municipal patient referral centre		Municipal referral centre (for some services)
	CRUE [Emergency Management Centre]	CRUE [Emergency Management Centre]			State patient referral centre (urgent care, hospital admissions and high technology tests)	State patient referral centre (urgent care, hospital admissions and high technology tests)	State referral centre (urgent care, hospital admittances and high technology tests)
	Informal communication (telephone/Internet) (specific processes)	Informal communication (telephone/Internet) (specific processes)	Informal communication (telephone/Internet)^b^	Informal communication (telephone/Internet)^b^	Informal communication (telephone)	Informal communication (telephone)	Informal communication (telephone)
	Permanent referral counter-referral committee (general population)	Permanent referral counter-referral committee (pregnancy and chronic)					

^a^ Used among ambulatory care centres and hospitals

^b^ Used only by primary care doctors and specialists within the ambulatory care centres and some specialists assigned to the network

### Opinions on the use of existing care coordination mechanisms in the healthcare networks

Although there are some differences between countries and networks, the informants’ discourse mainly highlights difficulties in the use of existing care coordination mechanisms, especially that of the health professionals. These difficulties vary depending on the mechanism and have important consequences for patient care.

### Mechanisms for information transfer across care levels

According to the majority of informants, the referral and counter-referral form (for transitions between primary and outpatient secondary care) and the discharge report (for transitions between hospital and primary care) are the mechanisms generally used to transfer clinical information between levels. In all of the networks, the user is responsible for transporting the documents.

In both countries, informants frequently report limitations in the use of the *referral and counter-referral forms*, although in Brazil fundamental problems emerge with more intensity, such as the absence of basic clinical information (e.g. the reason for the referral by primary care doctors) and the lack of counter-referrals by specialists. According to the majority, primary care doctors use the referral form mostly as an administrative mechanism to direct the patient to the required medical specialty area and not as a mechanism for sharing information: *“It is difficult when you get a referral form like this: vascular! That’s all! Where is the clinical information of the patient? And the exams? What medication has he been taking? It becomes quite difficult to proceed in these conditions”* (Secondary care professional, Network 3 Brazil).

In Colombia, however, the main problems highlighted were insufficient information recorded on medication and diagnostic tests in the referral form, and on the clinical management required in the counter-referral form, as well as the scarce use of the form on the part of some specialists (Table 4): *“In general the information is very summarized, the information that is sent in the counter-referral or referral [form] is very poor”* (Primary care professional, Network 3-C Colombia).

**Table 4:** Examples of the category opinion on “Information transfer mechanisms”

1. Referral and counter-referral forms
*“… We don’t have a counter-referral, we don’t know what occurred between* [the specialist] *and the patient, unless the patient tells us…”* (Primary care level professional, Network 1 Brazil)
*“In general they become involved in the bureaucratic function of the paper* [the referral form] *and very little is written down. Even when they have the paper available, many times there is very little information about the patient’s history… they don’t explain why the patients are being referred to the specialists or what has been done”* (Provider manager, Network 1 Brazil)
*“We have a referral system, the doctor refers* [the patient]*, explains everything, all of it, but when the time comes to counter-refer the specialists never counter-refer”* (Primary care level professional, Network 1-S Colombia)
*“The counter-referral works for us with AIDS, chronic conditions and pregnancies, because the operation of the program implies an administrative component that supports the doctor in this, do I explain myself? (…) so for a high risk pregnancy, the perinatalogist conducts a lengthy consultation…has a nurse to give admin support and they have a unified system that means that they don’t have to copy the same* [information] *three times…”* (Insurance manager, Network 2-S, Colombia)
2. Hospital discharge report
“*when the patient is hospitalized and leaves we always give him a copy of the discharge report, that is to say that the patient leaves with a copy of what he’s had done, (…)”* (Secondary care professional, Network 4-C Colombia)
*“We do use the hospital discharge report. When a patient is discharged the doctor prepares a summary of the problem and what procedures were performed. It’s a way for the professional in primary care to follow-up the patient.”* (Secondary care professional, Network 3 Brazil)
*“they are generally poorly filled out* [the discharge report]” (Primary care level professional, Network 1 Brazil)
*“there is always a discharge report if the doctor is resident, when the hospital has a resident doctor, because if it doesn’t…”* (Primary care level professional, Network 1 Brazil)
*“What we do fill out is the hospital admission form, make a summary of the clinical record, but in many cases the outpatient care management plan isn’t specified”* (Secondary care professional, Network 3-C Colombia)
3. Shared electronic clinical record
*“We do know what happens to the patient, because the patient continues in the system despite going to endocrinology…We use the same system, so we can consult the record of the sub-specialist, the specialist in the majority of cases, so we can see what the cardiologist said, what the endocrinologist said, what the neurologist said”* (Primary care professional, Network 3-C Colombia)
*“the quality of the clinical record is poor in general, right? There may be other good things to be said about it, but in general the quality of the clinical record is poor. The tool is good and it could be improved, but the quality is poor. The quality of the record, what professionals include there”* (Primary care level professional, Network 4-C Colombia)
*“one of the problems we have had is that the clinical records of the specialists are mostly incomplete compared to those of the paediatricians and general physicians”* (Provider manager, Network 3-C Colombia)
4. Problems with patient care due to lack of information transfer between levels
*“There are other things one doesn’t know…if it was a test for two or three months to see if it would work or not; or if the dosage was supposed to get progressively higher or lower. Many times the specialist does explain this to the patient, but you could say that there are patients who don’t understand the information very well, so you ask and since they are not given written information, you end up getting a bit lost”* (Primary care professional, Network 4 Colombia)
*“if he* [the primary care doctor] *doesn’t give anything back to me then I won’t know if he investigated because the patient doesn’t know whether the doctor investigated or not(…) So I won’t know and now what? I start again from zero. This creates problems, one gets trapped this way because I don’t know what I am going to do next or what was done or what I have to do now”* (Primary care professional, Network 3 Brazil)
*“it holds things up,… it reverts back to basic care, they* [the patients] *should arrive with a clinical record saying what they have so you can follow-up after that point. No!, when he* [the patient] *arrives he starts over at zero, you have to begin as though it were basic care (…) So it’s the patient who is most hurt by this process, because he wastes time trying to get exams done that he should have brought with him”* (Secondary care professional, Network 3 Brazil)
*“not having a shared clinical record affects us…many times exams are repeated unnecessarily, because in one institution they don’t know of all the exams carried out on a patient in another institution”* (Insurance manager, Network 1-S Colombia)

The clinical information transferred between the two care levels is only considered adequate in certain specific situations*:* in Colombia, in some specific District Health Department programs (AIDS, antenatal care, chronic pathologies) in the public providers of the subsidized network in Bogota, and in the ambulatory care centres of the contributory networks; and in Brazil, for some notifiable diseases (leprosy, tuberculosis). Furthermore, most informants consider that the transfer of clinical information to primary care is better following hospital discharge, and they stress that the discharge report is consistently delivered to the users, although there are some exceptions, according to primary care professionals. Nevertheless, while primary care professionals in Brazil consider that the information recorded in the discharge report is generally incomplete, in Colombia it is considered sufficient, except for the lack of instructions for care management plans.

Shared electronic medical records were only available in the ambulatory care centres of the contributory networks. Most health professionals believe that this mechanism improves information transfer between the primary care physician and the specialist, since it gives them permanent access to the patient’s clinical information. However, they also point out pitfalls: that they do not include information on hospital care and some external specialists, together with the insufficient recording of information on the part of specialists.

As a consequence of the deficiencies in the transfer of clinical information, the majority of professionals highlight the need to obtain information from the patients themselves on the care they have received, which generates errors as they are relying on the knowledge and understanding of the patients: “*The patient is in no condition to comment on what’s going on, he is not in the position, even if it has been clearly explained to him, he’s not a specialist in the health field, he doesn’t know the related jargon, he doesn’t know the procedures (…) he does not know how to interpret the exam”* (Primary care professional, Network 2 Brazil).

Thus the limited clinical information transfer between levels generates problems in care management for patients which vary according to the care level. Firstly, primary care professionals point to problems in the follow-up of patients due to having no definitive diagnosis, nor instructions for the correct treatment, thus resulting in interruptions in treatment and medical errors that lead to inappropriate and repeated referrals of the patient to outpatient secondary care and avoidable hospitalizations. Secondly, secondary care professionals point out the need to restart the diagnostic process, generating a delay in diagnosis and treatment, as well as greater healthcare expenses due to duplication of diagnostic tests: “*That is how it is, if this record was filled in correctly, I could attend to the patient and follow-up the patient here, and he would not necessarily have to go around consulting various specialists, and this would reduce the demand on specialists, you see?”* (Primary care level professional, Network 1 Brazil); *“(…) Well, they took the CAT, but when? Where? “No, I can’t remember”, and so one ends up saying “No, they don’t remember where it was done, so we have to start again”* (Secondary care professional, Network 3-C Colombia).

### Clinical management coordination mechanisms

Mechanisms for clinical management coordination are practically absent from the informants’ discourse in the networks studied. Almost exclusively in ambulatory care centres of the contributory networks, certain mechanisms for the standardization of skills (expert system: joint case review, shared clinical sessions, etc.) and of processes (clinical guidelines (CG)) emerge as the most frequently used mechanisms for the coordination of clinical management between the primary care doctor and the specialist. The joint review of cases also exists in one of the networks in Brazil (Recife), but according to informants, it is still in the initial phase of implementation. There are also clinical guidelines in the subsidized networks in Colombia, but only for specific processes such as maternal-perinatal care, and only for the public providers of the network. In all other cases, existing clinical protocols are not shared with other levels of care.

Informants from ambulatory care centres of the contributory networks highlight the existence of a group of specialists *(“Staff” or “primary groups”)–*generally referred to as the *expert system-*which lends support to primary care doctors through periodic joint clinical sessions for the review of cases or topics, joint appointments with patients and a permanent consultation service both in person and by phone. Informants of the Recife network in Brazil also mention the recent implementation of a Health Department support program (*“apoio matricial”),* but this is only for the discussion of cases and topics of certain medical specialties, and it primarily depends on the support of specialists that do not work in the network. The informants consider that the expert system favours communication between professionals, improves the capacity of primary care to resolve health problems, and helps to define criteria for the correct referral of patients. However, some professionals identify problems with its use: the most common complaint is that there are simply not enough opportunities for professionals to liaise; in the contributory networks, some important medical specialties are excluded from the program because the hospitals of the network are not involved; and, in the Brazilian network, participation levels are low because the sessions take place during consultation hours and specialists who do not work in the network are not remunerated due to insufficient funding of the program (Table 5): *“There’s no way we can do any more, because if every time you have a meeting you have to pull teams away from the unit, someone is not providing care, and it is pretty difficult to do”* (Provider manager, network 1 Brazil).

**Table 5:** Examples of the category “Clinical management coordination mechanisms”

1. Benefits of the expert system
*“There’s the Staff* [specialist support groups] *with their different specialties, we in the ambulatory care centre have Gynaecology, Paediatrics, Psychology, Orthopaedics, Internal Medicine and… that’s all the Staff we have. In each meeting we review cases and the specialists help us to resolve the cases”* (Primary care professional, Network 4-C)
*“The question of the “matriciamento”* [expert system], *is what I was telling you about, the question of maintaining this communication with the secondary care network, tertiary care, and managing to resolve cases that primary care can’t resolve in an effective way, understand? Because the patient comes back to you and you conclude his treatment here, but now with the specialist’s opinion; I think that helps to resolve cases”* (Primary care professional, Network 1 Brazil).
2. Problems with the expert system
*“the time we have to interact is really very little, that’s why I say that this space here, one hour a month is very important, but it’s not enough”* (Primary care professional, Network 4-C)
*“this is done only once a month* [expert system],*” because people can’t do any more, because every time you hold a meeting you take teams from the unit, someone is not providing care, it is very difficult to do* (Provider manager Network 1 Brazil)
3. Low adherence to the CPG
*“(…) the dynamics of the rotation of professionals influences the level of adherence to the guidelines and protocols. When we evaluate we find everything, professionals who stick closely to the guidelines and, more often than not, others who don’t, but it all comes down to the constant changes of personnel” (Provider manager,* Network 2-S Colombia*)*
*“The hospitals do not adhere to the guidelines, we proved this in the last network committee, about a maternal death in a third level hospital, the guidelines themselves were not taken into account for the care of the patient, it’s a lack of adherence to guidelines”* (Administrative insurance professional, Network 2-S Colombia)

With regard to clinical guidelines, most ambulatory care centre managers and professionals in the contributory and subsidized networks believe that they contribute to care coordination between professionals of different care levels because they serve to direct the clinical management of pathologies and define the criteria for the referral of patients. However, many of the professionals interviewed pointed out that their use was limited due to difficulties such as insufficient time for review and consultation, lack of awareness of their existence and, in the subsidized networks, the scant commitment of insurers and providers to their implementation. *“Professionals should follow the guidelines, but they never showed them to us. (…) Guidelines? What guidelines? We haven’t been given any guidelines”* (Primary care level professional, Network 4-C Colombia).

### Administrative coordination mechanisms for access across care levels

According to informants, the most commonly used mechanisms in all of the networks for the administrative coordination of patient access between care levels are the *patient referral centres* (“*central de regulação*” in Brazil), which act as liaison hubs: in most of the Colombian networks they deal with urgent patient referrals and unprogrammed hospital admissions, and in Brazil they also manage specialist outpatient care. In Brazil, according to the informants, there are various patient referral centres within a single network which are dependent on the municipal and state health departments, in order to coordinate access to the services managed by the corresponding level of government.

Opinions on their effectiveness are somewhat conflicting in the two countries: informants in Brazil mainly reported problems, whereas most managers in Colombia considered them a facilitator for coordination as they contribute to a reduction in waiting times for urgent referrals. However, some professionals from one of the contributory networks pointed to long waiting times for urgent referrals due to the location of the insurer’s patient referral centre in another city and the limited awareness of the services available in Bogota.

According to informants of the networks analyzed in Brazil, there are several problems with the use of this mechanism (Table 6). First, the exclusion of high technology health services from the coordination, as well as the exclusion of the population from other municipalities-also covered by the network-who are forced to personally search out a facility to receive care and to manage their own care program, “*the diabetic patient, he has a foot lesion and needs an opinion from a vascular surgeon (…) To do this, this referral, I believe what happens today is what happens in the majority of cases, right? The sick patient has to find services on his own”* (Primary care professional, Network 2 Brazil). Second, the referral of urgent patients to inappropriate health facilities-for example, to hospitals that have previously notified the unavailability of beds or personnel, to health units not accredited to care for that condition etc.-without a subsequent follow-up of the referral process in order to refer the patient to other units if they are not attended to: *“They* [the state patient referral centre] *do not know what type of obstetrics care you can receive here. So many times a patient arrives with a high-risk pregnancy and clear indications that she should have her baby in a hospital that has a neonatal intensive care unit, because there is a risk to the baby, and the patient comes here. I can’t refuse to treat her, nor can I keep her here because it may put her life or the baby’s life at risk”* (Provider manager, Network 2 Brazil). Third, long waiting times in the referral of urgent patients to outpatient secondary care and for diagnostic tests. Fourth, inappropriate programming-in terms of timing-of outpatient secondary services, because the system does not take into account the patient’s ability to keep the appointment, resulting in many missed appointments. Lastly, the lack of collaboration between the various referral centres that operate in the network, which hinders the referral and counter-referral of patients between services coordinated by different referral centres.

**Table 6:** Examples of the category “Administrative coordination mechanisms”

Patient referral centres
*“There are a lot of delays in referrals, because they call another city, they call the provider, because they serve as the intermediary between the two parts, and this takes a long time*” (Administrative professional secondary care, Network 4-C Colombia).
*“it’s the story of the UTI* [Intensive Care Unit]*, it takes 2,3,4 days to get a bed, if he* [the patient] *survives or gets better, good, if not….. So the problem is really very grave in this sense”* (Provider manager, Network 2 Brazil)
*“our information system has this fault, it doesn’t allow us to confirm the scheduling of the patient’s appointment, much less whether the appointment actually took place. So while all this is going on,(…) the professional ends up with various gaps in their appointments schedule (…) when I have a huge demand from patients waiting to be seen who could have been rescheduled to fill the gaps…”.* (Provider manager, Network 3 Brazil)
*“I know that this strategy of jumping the queue happens, because I’ve seen it in other services and it’s the same thing. But if we were to carefully analyze this practice, we would see that it is unjust, because if I don’t know anyone who can help me get my patient seen, then he’s going to have to wait in line, isn’t he.”* (Primary care professional, Network 2 Brazil)

Informants of the Brazilian networks point out that, as a consequence of the lack of administrative coordination in access, there is an extensive use of informal networks (based on personal contacts) to identify the appropriate service within the network to which to refer the patient or to get more rapid access to a health service (beds, visits, diagnostic tests, etc.). Nevertheless, they believe that this strategy generates inequities because access is related to factors other than need, such as the interests of health professionals, their informal networks or political influence, or the information and contacts of the patient. *“These days we get many things done through informal means. To get a hospital bed, people don’t tend to go through the patient referral centre, people pull strings because they know someone from Hospital XX, because they know through Hospital YYY that such and such is working at some hospital and they can get a bed there (…) basically, people get it informally, but they shouldn’t, right?”* (Secondary care professional, Network 1 Brazil).

In the subsidized networks in Colombia, informants identified the use of other mechanisms for coordinating patient access, such as informal communication between professionals (telephone, email, internet) for the referral of patients to emergency care in specific processes (maternal-perinatal and uterine cancer) or complex cases, and permanent committees to improve the administrative coordination of patient referral and counter-referral between public healthcare providers. A number of difficulties in the use of both emerged: in the case of informal communication mechanisms, there were technical problems with phone and internet services among primary care providers, and in that of the permanent committees, the profiles of the members were inadequate, participation was erratic and the providers’ managers were unwilling to address the problems detected in the committees.

## Discussion

Few studies have analyzed the experience of health personnel in the use of care coordination mechanisms implemented in healthcare networks, covering the different dimensions of coordination with a broad organizational overview, rather than focusing on a specific program or mechanism [2, 17, 18]. In fact, in the Latin American context, analyses of integrated healthcare networks are practically non-existent [42]. This exploratory qualitative study contributes to current knowledge through a series of healthcare network case studies that provide information regarding the limitations and benefits of the use of coordination mechanisms and the implications for patient care from the perspective of the health personnel involved. It does not aim to generalize the results from a statistically representative sample, but rather from the process of generation of ideas that stem from the specificities of concrete cases [51]. Furthermore, it is a transnational comparative study, which allows us to draw conclusions on similarities and differences in the use of the mechanisms in health systems and networks with different organizational models.

Despite the differences between countries and networks, the results of this study show that coordination mechanisms are poorly implemented in the majority of the networks studied. There are exceptions to this in specific parts of the networks: in Colombia, in the contributory network ambulatory care centres linked to the insurer through a merger or strategic alliance, and among the public providers of the subsidized network managed by the District Health Department; and in Brazil, among some of the facilities in the network of the state capital. These results highlight the difficulties involved in implementing coordination mechanisms that take in the whole network in a health system model such as that of Colombia, which incentivizes networks that foster competition between providers, the fragmentation of care provision into multiple providers and instability in working relationships due to the preponderance of short-term contracts with providers. We can add to this the inefficiency of the health authority–in this case, the Health Department-in getting providers and private insurers of the subsidized scheme involved in the implementation of the interventions developed by public providers [52, 53]. In Brazil, the smallest municipalities encounter difficulties, as they lack teams with the technical qualifications needed to implant care coordination mechanisms in the healthcare networks for their population [10, 54, 55].

Most informants, in particular health professionals, also identify various pitfalls in the use of existing mechanisms, mostly related to information and administrative coordination, which have significant repercussions on patient care. The only informants to acknowledge the contributions of certain mechanisms to care coordination were from ambulatory care centres of the contributory networks, but they also point out pitfalls in their use.

### Difficulties in the transfer of patient information across care levels and the negative impact on efficiency and quality of care

The results show weaknesses in the transfer of clinical information across care levels in the networks analyzed due to insufficient recording of data on the part of health professionals, especially in the transfer from primary care to outpatient secondary care. This is a problem often cited in other studies, mainly carried out in the health systems of high income countries [17, 56–59] but also in some middle and low-income countries [60, 61], and is usually related to the lack of time for recording information [56]. In the context of this study, in addition to the lack of time, it is related to the lack of willingness to communicate between levels, especially on the part of the secondary care professionals, due to a lack of interest, awareness, appreciation and trust in the role of the primary care level in patient follow-ups [53]. These factors seem to be related to working conditions (precarious working conditions, fee-for-service reimbursement etc.) and with the inadequate training of professionals (high specialization in medicine, lack of ongoing training). Therefore strategies should be implemented to address these factors in order to improve the use of existing referral and counter-referral forms by professionals, before implementing mechanisms that require a greater investment, such as those based on information technology (shared medical records, etc.) [58], systems in which, as informants pointed out, similar problems arise with the quality of data recording.

In agreement with other studies, the informants highlight the costs incurred by inadequate information transfer between levels-avoidable referrals and hospitalizations, duplication of tests-[17] and the impact on the quality of care and health of the patients due to delays in diagnosis and interruptions in treatment [58, 59]. It also poses an obstacle to the correct follow-up of patients by primary care doctors, which also has negative repercussions on the quality and costs of care.

Furthermore, patients become intermediaries in the transfer of information, a factor which, according to the professionals interviewed, can produce significant medical errors. According to other studies, this generates discomfort among users-who have to take on a responsibility that they should not have to assume-and also distrust in the quality of care received [62].

### Scarce development of mechanisms that favour clinical management coordination

The results show the limited development of mechanisms for the coordination of clinical management-with the exception of the ambulatory care centres of the contributory networks-in spite of its central importance to care coordination. Furthermore, the mechanisms identified have been introduced in an isolated way and do not form part of shared care strategies that make use of various different instruments simultaneously and create personalized care plans for the patient. According to the literature, these characteristics contribute to the greater effectiveness of strategies in addressing chronic health problems [17, 63–65].

The expert system and shared clinical guidelines are the mechanisms used for clinical management coordination. The former is especially valued by the professionals in the networks that have implemented it, not only for improving the resolution capacity of the primary care level and the adequacy of referrals, but also for creating spaces that favour communication among professionals, a point in keeping with other studies [66]. The role of specialists within the network in providing support to primary care doctors is key to the successful implementation of this and other strategies for improving coordination [19]. This presents an argument against the participation of specialists from outside the network in the type of expert system (“apoio matricial”) that the Brazilian networks are trying to implement.

The professionals point out the potential benefits of *shared clinical guidelines* for improving the quality of referrals, but they stress that there is little adherence to the guidelines. Studies conducted in other settings usually relate these low levels of use not only to the limited reach of the implementation process that results in a lack of awareness of the mechanism, but also to the scarce participation of health professionals in the design of the mechanism itself [19, 24, 27, 67], and its lack of translation into protocols and incorporation into ongoing training programs [68]. The results of this study also reveal the significant influence of the working conditions of the professionals using the mechanisms, such as the frequent rotation of personnel that results in lack of awareness, and the limited time they have available to make use of the mechanisms.

### Difficulties in administrative coordination of access of the patient referral centres

The results also reveal significant problems with the use of referral centres, the principal mechanism for the administrative coordination of access for patients, such as the lack of referral or inappropriate referral of patients, especially in the Brazilian networks. As well as being related to the limitations of the mechanism itself, these problems are also associated with other elements of the health system, such as the presence of structural barriers to access and the fragmentation of care in a decentralized healthcare model. The former contributes to referral centres failing to refer patients to the right location at the right time due to the lack of services available, and the latter, to the presence of multiple actors (state, municipal) with incentives to compete for resources instead of collaborating with each other [10] to coordinate the various patient referral centres in the network.

These limitations are so great that they lead to health professionals and patients making frequent use of informal networks to access health services, an unfortunate alternative to the institutional mechanism, as most informants point out, due to its grave consequences for equity in access to care.

### Lessons for the use of coordination mechanisms from the experiences of Colombia and Brazil

Various lessons can be drawn from both the difficulties cited in the use of coordination mechanisms and certain contributions identified by the informants that might be relevant for policy makers, managers and professionals of health networks in these and other contexts. Firstly, there should be greater efforts to encourage the effective implementation of new care coordination mechanisms, especially those directed at care management. This requires generating the right conditions for their use. The same is true for existing mechanisms, which in some cases are sufficient to improve coordination, such as the correct use of referral and counter-referral forms and patient referral centres. Achieving these conditions may require the introduction of organizational changes in the networks (adequate working conditions or payment methods for professionals that provide an incentive to cooperate), the promotion of values and positive attitudes to collaboration, or substantial modifications in the organization of the healthcare system to reduce network fragmentation. Secondly, it is clear that the implementation administrative coordination mechanisms of access across levels of care (patient referral centres etc.), which could contribute to patients receiving care at the most appropriate level, does not resolve access problems when their causes are of a structural nature. Finally, another lesson to be gained is the relevance of implementing mutual adjustment mechanisms. In keeping with other studies, our results demonstrate that despite the tendency of the managers of organizations to implement mechanisms based on the standardization of work processes, professionals value more highly those mechanisms that create spaces for direct communication, not only because they are more efficient in coordinating complex processes, but also because they improve shared knowledge and interpersonal relationships, which are the foundations for encouraging collaborative attitudes.

### Limitations of the study

In Colombia, most of the insurers operating in the study area refused to participate. This may lead us to expect that the four networks that were finally selected perform better. However, the results indicate significant difficulties in the implementation of coordination mechanisms in these networks. The lack of previous studies or statistical information on care coordination in Colombia makes it difficult to contrast the results with other studies. These characteristics should be taken into account in the interpretation of results and their transferral to other contexts.

## Conclusions

This study shows not only the limited development of mechanisms for care coordination across levels of care in the healthcare networks analyzed in Colombia and Brazil, but also a limited use of existing mechanisms, with an ensuing perceived negative impact on coordination, efficiency and quality of care. A lesson for these and other contexts is that it is equally important to develop mechanisms for improving coordination in the networks-especially those aimed at clinical care management and based on mutual adjustment-as it is to ensure the proper conditions for their use, which may require organizational changes in networks and healthcare systems.

## Abbreviations

IHN: Integrated healthcare networks
SGSSS: General system of social security in health
EPS-S: Insurance company for the subsidized regime
EPS: Insurance company for the contributory regime
PSF: Family health program
